# Altered intestinal functions and increased local inflammation in insulin-resistant obese subjects: a gene-expression profile analysis

**DOI:** 10.1186/s12876-015-0342-y

**Published:** 2015-09-16

**Authors:** Alain Veilleux, Sylvain Mayeur, Jean-Christophe Bérubé, Jean-François Beaulieu, Eric Tremblay, Frédéric-Simon Hould, Yohan Bossé, Denis Richard, Emile Levy

**Affiliations:** 1 Department of Nutrition, Université de Montréal and Research center of CHU Sainte-Justine, 3175 Côte Ste-Catherine, Montréal, Qc Canada; 2 Institut universitaire de cardiologie et de pneumologie de Québec (CRIUCPQ), Université Laval, Québec, Qc Canada; 3 Departement of Anatomy and cellular biology, Université de Sherbrooke, Sherbrooke, Qc Canada; 4 Canada Research Chair in Intestinal Physiopathology, Sherbrooke, Québec Canada; 5 Departement of surgery, Université Laval, Québec, Qc Canada; 6 Department of Molecular Medicine, Université Laval, Quebec, Qc Canada; 7 Chaire de Recherche Merck Frosst/IRSC Research Chair on Obesity, Québec, Qc Canada; 8 JA. deSève Research Chair in nutrition, Montréal, Qc Canada

## Abstract

**Background:**

Metabolic alterations relevant to postprandial dyslipidemia were previously identified in the intestine of obese insulin-resistant subjects. The aim of the study was to identify the genes deregulated by systemic insulin resistance in the intestine of severely obese subjects.

**Methods:**

Transcripts from duodenal samples of insulin-sensitive (HOMA-IR < 3, *n* = 9) and insulin-resistant (HOMA-IR > 7, *n* = 9) obese subjects were assayed by microarray (Illumina HumanHT-12).

**Results:**

A total of 195 annotated genes were identified as differentially expressed between these two groups (Fold change > 1.2). Of these genes, 36 were found to be directly involved in known intestinal functions, including digestion, extracellular matrix, endocrine system, immunity and cholesterol metabolism. Interestingly, all differentially expressed genes (*n* = 8) implicated in inflammation and oxidative stress were found to be upregulated in the intestine of insulin-resistant compared to insulin-sensitive subjects. Metabolic pathway analysis revealed that several signaling pathways involved in immunity and inflammation were significantly enriched in differently expressed genes and were predicted to be activated in the intestine of insulin-resistant subjects. Using stringent criteria (Fold change > 1.5; FDR < 0.05), three genes were found to be significantly and differently expressed in the intestine of insulin-resistant compared to insulin-sensitive subjects: the transcripts of the insulinotropic glucose-dependant peptide (*GIP*) and of the β-microseminoprotein (*MSMB*) were significantly reduced, but that of the humanin like-1 (*MTRNR2L1*) was significantly increased.

**Conclusion:**

These results underline that systemic insulin resistance is associated with remodeling of key intestinal functions. Moreover, these data indicate that small intestine metabolic dysfunction is accompanied with a local amplification of low-grade inflammatory process implicating several pathways. Genes identified in this study are potentially triggered throughout the development of intestinal metabolic abnormalities, which could contribute to dyslipidemia, a component of metabolic syndrome and diabetes.

**Electronic supplementary material:**

The online version of this article (doi:10.1186/s12876-015-0342-y) contains supplementary material, which is available to authorized users.

## Background

Insulin resistance and type 2 diabetes (T2D) are frequently associated with fasting and postprandial dyslipidemia. Exaggerated hepatic VLDL production and reduced hepatic triglyceride-rich lipoprotein (TRL) clearance have long been suggested as the main mechanisms involved in this complication. However, growing evidence suggests that postprandial and fasting plasma lipids are regulated by an interaction between hepatic and intestinal metabolism [1]. In particular, the gut epithelium is well equipped to efficiently transform and transport alimentary lipids by employing competent lipoprotein assembly in synchronization with the whole-body homeostasis [2]. Postprandial overproduction of triglyceride-rich lipoproteins (TRL), mostly chylomicrons (CMs), have been associated to postprandial dyslipidemia in several well-designed human studies [1]. Clearly, development of insulin resistance and T2D has been linked to alterations in key intestinal metabolic functions [3–5].

Various animal models have been employed to examine the impact of systemic insulin resistance on intestinal lipid metabolism [3–9]. Most of the studies evidently report the role of local insulin resistance in promoting lipid metabolism dysregulation. However, only limited investigations have been performed in humans. Recently, our group [10] along with other laboratories [11, 12] have tested this hypothesis in insulin-resistant and hyperlipidemic subjects. As observed in other metabolic organs (i.e., liver, adipose tissue and muscle), the small intestine of insulin-resistant subjects displayed evidence of insulin signaling defects [10]. As stressed by our findings, local insulin resistance was likely the result of an exacerbated pro-oxidative and pro-inflammatory local environment, which resulted in enhanced *de novo* lipogenesis rate and apolipoprotein (apo) B48 biogenesis along with exaggerated TRL production and secretion [10]. Presence of oxidative stress in the intestine of insulin-resistant subjects was evidenced by increased malondialdehyde and conjugated dienes levels and by a decreased superoxide dismutase (SOD) antioxidant activity [10]. Conversely, the low grade inflammatory state was highlighted by enhanced p38 MAPK and NF-κB pathway activation and by increased expression levels of TNF-α, IL-6, ICAM and PTGS-2 in the intestine of insulin-resistant subjects [10]. Adverse changes in the expression of key proteins involved in lipid/lipoprotein metabolism [i.e., liver and intestinal specific fatty acid binding proteins (FABP), microsomal triglyceride transfer protein (MTP)] and cholesterol metabolism [i.e., adenosine triphosphatase-binding cassette A1 (ABCA1) and proprotein convertase subtilisin/kexin type 9 (PCSK9)] were also observed in insulin-resistant subjects [10].

Although dysfunctional intestinal lipid metabolism and concomitant postprandial TRL overproduction seem to pave the way to the development of atherogenic dyslipidemia and increased risks of cardiovascular diseases [13], the intra-enterocyte signalling pathways and intrinsic mechanisms, likely orchestrated by additional critical proteins, have remained obscure. Therefore, our objective in the present study was to delineate potential mechanisms and unravel new gene candidates using whole-genome gene expression microarray, one of the most powerful tools that allows the measurement of all known genes simultaneously and has been used extensively in biomedical researches. Our experimental approach has directly focused on human intestine to avoid limitations inherent to animal models. Taking advantage of the unique opportunity to obtain small intestine samples following bariatric surgery, we established tissue-specific gene expression signatures that characterized insulin-resistant and insulin-sensitive obese subjects.

## Methods

### Subjects and tissue sampling

Twenty severely obese subjects (BMI ≥ 40 kg/m^2^), without T2D according to their medical files and undergoing biliopancreatic diversion, were recruited at the Quebec Heart and Lung Institute*,* Laval University (Quebec City, Canada). Insulin and glucose levels were assessed in the plasma of each subject to calculate the homeostatic model assessment of insulin resistance (HOMA-IR) index. These subjects (10 women and 10 men) were assigned into two groups matched for sex, age (±10 years) and body mass index (BMI) (±5 units), but with either relatively low (<3) or high (>7) HOMA-IR. None of these subjects received a therapy with hypoglycaemic agents, cholesterol- or TG-lowering agents. Duodenum specimens were obtained during the surgery. The small intestine samples were immediately washed and frozen in liquid nitrogen and stored at −80 °C for subsequent RNA extraction. Written informed consent was obtained from all subjects. The project was approved by the ethics committees of Quebec Heart and Lung Institute (Quebec City, Canada) and Sainte-Justine Research Center (Montreal, Canada).

### Anthropometry, lipid profile and glucose homeostasis

BMI and overnight fasting blood samples were obtained in the morning before the surgery. Total cholesterol, free cholesterol and triglyceride levels were estimated in the plasma by colorimetric enzymatic kits (Roche Diagnostic, Indianapolis, USA). Glucose was assessed using the glucose oxidase method and insulin was quantified with the ultrasensitive insulin assay on the Access® immunoassay system (Beckman Coulter, Brea, USA). The HOMA-IR index was calculated using the following formula: fasting insulin (μU/mL) × fasting glucose (mmol/L)/22.5 [14].

### Intestinal Caco-2/15 cell culture

For functional studies, we used the intestinal Caco-2/15 cells [15], generated from a clone of the original Caco-2 (ATCC, Rockville, USA), were cultured at sub-confluence in MEM supplemented with 5 % fetal bovine serum, 1 % non-essential amino acids and 1 % Penicillin/Streptomycin (Gibco, Grand Island, USA) as previously described [15]. For individual experiments, cells were plated at a density of 5 × 10^5^ cells/well in flat-bottom 6-well plates (Costar, Cambridge, MA). Cells were cultured for 12–15 days post-confluence, which represents an appropriate period to reach full differentiation and study lipid and lipoprotein metabolism as well as oxidative stress and inflammation [16]. Cells were serum starved for 18 h and then incubated in presence or absence of iron-ascorbate (Fe/Asc) (200 μM/2 mM) for 6 h or LPS (150 μg/mL) for 24 h at 37 °C.

### Messenger RNA preparation

Total RNA was isolated from intestinal tissue using the RNeasy lipid tissue extraction kit and on-column DNase treatment following the manufacturer’s recommendations (Qiagen, Valencia, USA). RNA concentration and quality were respectively assessed using the NanoDrop spectrophotometer (Thermo Scientific, Wilmington, USA) and the Agilent Technologies 2100 bioanalyzer (Agilent, Santa Clara, USA). Only total RNA samples with a RNA integrity number higher than seven were included in the following experiments.

### Gene expression profile analysis

Microarray experiments were performed at the McGill University and Génome Québec Innovation Centre (Montréal, Canada). Gene expression profile was assessed using the Illumina HumanHT-12 v4 BeadChip (Illumina, San Diego, USA) technology. Briefly, reverse transcription of 250 ng total RNA and complementary RNA synthesis and biotin labeling were performed using the TargetAmp Labeling Kit (Illumina, San Diego, USA). Hybridization of 700 ng of each biotinylated cRNA sample on the BeadChip was performed at 58 °C for 16–18 h. BeadChips were scanned using the Illumina Bead Array Reader (Illumina, San Diego, USA). Main observations of the microarray experiments were validated by quantitative real-time PCR (qPCR). Complimentary DNA was generated using the superscript first strand synthesis system (Invitrogen, Carlsbad, USA). Real-time cDNA amplification was performed in triplicate using SYBR Green with the 7500 Real-Time PCR System (Applied Biosystems, Foster City, USA) for 40 cycles. Target gene amplifications were normalized to ATP synthase 5 subunit O (ATP5O) expression levels. The relative mRNA fold changes (insulin-resistant compared to insulin-sensitive subjects) were calculated using the 2^−ΔΔCt^ method. Validation was performed to ensure optimal cDNA amplification efficiency of the target and reference genes. Primer sequences used for PCR were as follows (sense; anti-sense): *GIP* (TGGCAGTGGGACTAGGAGAG; GTTGAGGGCTGCTCACCTTA), *MSMB* (GCTTTGCCTGGATGTACCTG; CCTGGTTGAATCTCCTGGAA), MT*RNR2L1* (CACTTGTTCCTTAAATAGGGACTTGTC; AGCTGAACCCTCGTGGAGC) and *ATP5O* (GCGATGCTTCAGTACCTCTG; TGGCATAGCGACCTTCAATA).

### Statistical analyses

Differences in metabolic parameters between insulin-sensitive and insulin-resistant were tested using paired Student’s *t*-test and repeated measures analysis of variances (ANOVA). Microarray expression data were extracted using the Robust Multichip Average (RMA) method [17] and were analyzed using R. Prior to microarray analysis, one pair of male subjects was removed as one sample failed the quality control and was considered as an outlier using the distance distribution clustering analysis. Differentially expressed genes were identified as having an estimated fold-change of at least 1.5 (intestine of insulin-resistant subjects compared to that of insulin-sensitive subjects) and a false discovery rate (FDR) smaller than 0.05 was calculated using the Significant Analysis of Microarrays method [18]. In addition to this stringent primary analysis, a second set of differentially expressed genes were identified using more permissive analysis parameters to perform pathway analysis [19, 20]. A total of 195 genes with a fold change greater than 1.2 and with a *p* value smaller than 0.05 were included in the metabolic pathway analysis [Ingenuity Pathway Analysis (IPA) software, Qiagen, Redwood City, USA]. Gene Ontology (GO) enrichment analysis, including p-values for all genes present on the array, was carried out using GOrilla (http://cbl-gorilla.cs.technion.ac.il/). Briefly, the 47 313 probes were ranked according to their absolute fold change and mapped to the 36 683 corresponding genes in the GOrilla system. After extraction of the highest ranking instance of duplicates, 21 918 genes were used for testing. A total of 17 978 of these genes were associated with a gene ontology biological process terms. GO terms with enrichment p-value lower than 10^−5^ were considered. Messenger RNA expression levels (ΔΔCt) measured by qPCR were compared using the Wilcoxson signed-rank test or the Student’s *t*-test, respectively, for experiments including human samples and Caco-2/15 cells. Spearman correlation coefficients were computed to quantify the association between specific microarray data and qPCR expression values. Differences were considered to be statistically significant at *p* < 0.05. Statistical analyses were performed with SAS software (SAS Institute, Cary, USA).

## Results

**Fig. 1:**
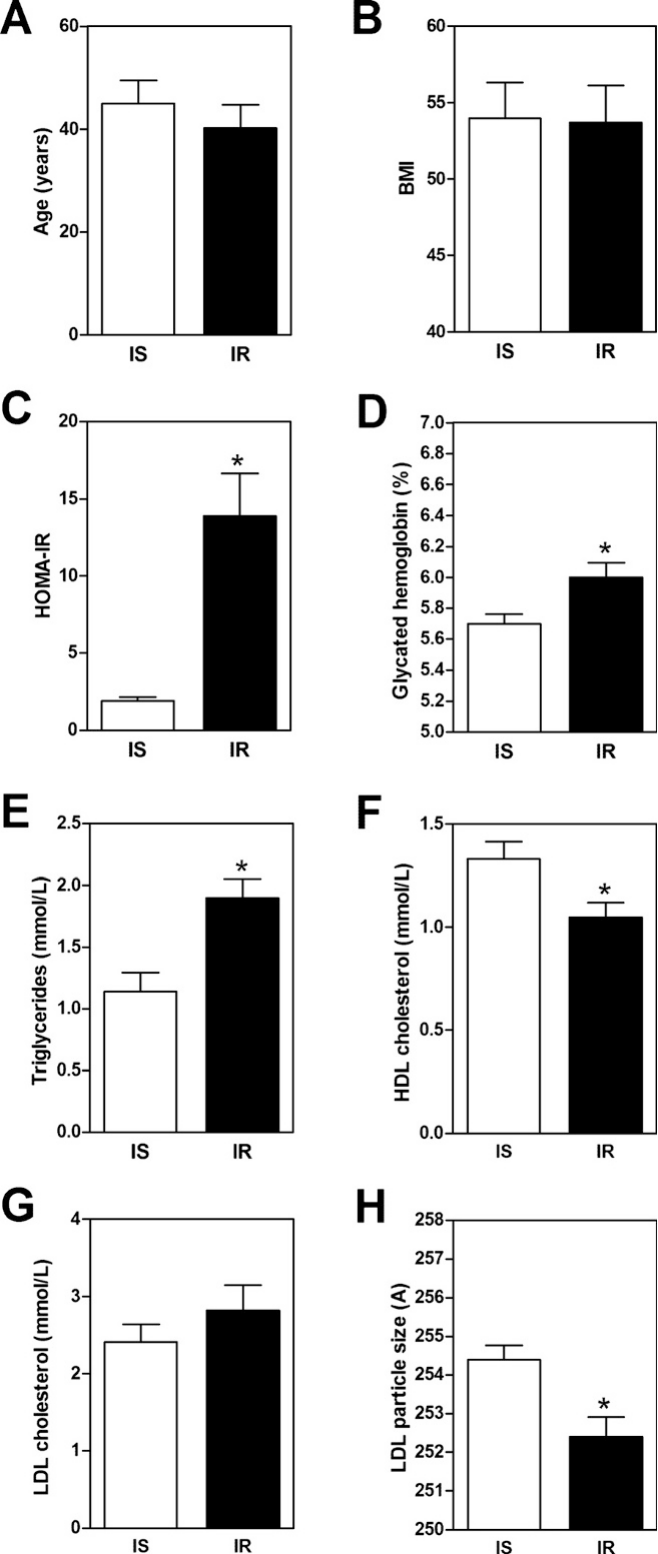
Anthropometric, glucose homeostasis and lipid profile parameters in insulin-sensitive (*n* = 9) and insulin-resistant subjects (*n* = 9). Overnight fast values of **a** age, **b** BMI, **c** HOMA-IR index, **d** glycosylated hemoglobin (%), **e)** plasma TG (mmol/L), **f** HDL cholesterol (mmol/L), **g** LDL cholesterol (mmol/L), and **h** LDL particle size are shown. Values are mean ± SEM

Messenger RNA expression was measured in the intestine of 18 severely obese women (*n* = 10) and men (*n* = 8). All subjects were candidates for bariatric surgery, and specimens of the proximal small intestine were obtained during the procedure. Subjects were 42.6 years on average and had a mean BMI of 53.8 kg/m^2^ [10]. The study sample was selected from a larger pool to establish two groups paired for sex, age and BMI but with divergent HOMA-IR index values, namely the insulin-sensitive (HOMA-IR < 3, *n* = 9) and the insulin-resistant (HOMA-IR > 7, *n* = 9) obese subjects [10]. No differences in age (45.0 ± 10.0 vs. 40.3 ± 10.1 years) and BMI (54.0 ± 7.3 vs. 53.7 ± 7.6 kg/m^2^) were observed between the two groups (Fig. 1a, b). Nonetheless, insulin-resistant subjects were characterized by a higher HOMA-IR index and elevated glycosylated hemoglobin, which is in line with the study design (Fig. 1c, d). They also present a less favorable fasting plasma lipid profile characterized by higher triglyceride (TG) and lower HDL-cholesterol concentrations, as well as smaller LDL particles (Fig.1 e–h).

Microarray analysis of duodenal samples from insulin-sensitive (*n* = 9) and insulin-resistant (*n* = 9) obese subjects reveals a relatively low intergroup variability in gene expression profiles. A total of 195 annotated genes were identified in the intestine of these subjects using relatively permissive analysis parameters (fold change > 1.2 and p value < 0.05) [19, 20]. The complete list of differentially expressed genes are included in the [see Additional file 1 : Table S1]. Of these differentially expressed genes, 36 were known to be involved in different small intestine functions such as signaling peptides, inflammation, oxidative stress, nutrient transport, extracellular matrix, digestive functions, immunity and cholesterol homeostasis (Table 1). These results pointed out the presence of alterations in several key intestinal functions in presence of systemic insulin resistance. Some interesting trends were noted. Expression levels of several genes relevant to intestinal extracellular matrix, including those coding for mucin proteins as well as for metalloproteinase 7 and villin 1, were altered in presence of insulin resistance (Table 1). Expression levels of enzymes involved in a number of digestive steps (Pepsinogen A, lactase, β-galactosidase, pancreatic lipase, trypsin 1 and elastase) were also modified and may collectively denote changes in the interaction between the gut and the alimentary and metabolic status. It is also noteworthy that all differentially expressed genes involved in inflammation and oxidative stress pathways (*PTGS2, DUSD1, S100A8, S100A9, IL1B, GPX8*, *FOS* and *JUN*) were found to be upregulated in insulin-resistant subjects. Finally, we have found that several major histocompatibility complex members were differentially expressed in the intestine of insulin-sensitive and insulin-resistant obese subjects.

**Table 1:** Differentially expressed genes in the intestine of insulin-resistant compared to insulin-sensitive subjects

Function	Genes		Fold change
Intestinal peptides	GIP	Glucose inhibitory peptide	−1.72
	GKN1	Gastrokine 1	−1.54
	GKN2	Gastrokine 2	−1.51
	VIP	Vasoactive intestinal polypeptide	1.30
Inflammation and oxidative stress	PTGS2	Prostaglandin-endoperoxide synthase – COX-2	1.20
	DUSD1	Dual specificity phosphatase 1	1.37
	S100A8	S100 calcium binding protein A8	1.59
	S100A9	S100 calcium binding protein A9	1.51
	IL1B	Interleukin 1 beta	1.21
	GPX8	Glutathione peroxidase 8	1.21
	FOS	Cellular oncogene c-Fos	1.55
	JUN	Jun proto-oncogene	1.32
Transporters	SLC28A2	Sodium-coupled nucleoside transporter	−1.68
	SLC2A3	Facilitated glucose transporter 3	1.25
Extracellular matrix	MUC5AC	Mucin 5 AC	−1.58
	MUC2	Mucin 2	−1.32
	MUC6	Mucin 6	1.21
	MMP7	Metalloproteinase 7	1.27
	VIL1	Villin 1	−1.22
Digestive functions	PGA5	Pepsinogen A	−1.97
	LCT	Lactase	1.76
	GLB1	β-galactosidase	−1.21
	PNLIPRP2	Pancreatic lipase	1.30
	PRSS1	Trypsin 1	1.24
	ELA3A	Elastase	1.25
Immunity	GP2	Glycoprotein 2	1.38
	HLA-A	Major histocompatibility complex class I A	2.63
	HLA-C	Major histocompatibility complex class I C	−1.20
	HLA-F	Major histocompatibility complex class I F	−1.28
	HLA-DQA1	Major histocompatibility complex class II DQ alpha 1	1.38
	HLA-DQB1	Major histocompatibility complex class II DQ beta 1	1.22
	HLA-DRB1	Major histocompatibility complex class II DR beta 1	−1.35
	HLA-DRB4	Major histocompatibility complex class II DR beta 4	1.39
Cholesterol homeostasis	LDLR	Low density lipoprotein receptor	1.30
	APOD	Apolipoprotein D-III	1.20
	APOA1	Apolipoprotein A-I	−1.37

Subsequently, the 195 differentially expressed genes in the intestine were investigated using the Ingenuity Pathway Analysis (IPA) software. This approach allowed the identification of 17 metabolic pathways significantly enriched in differentially expressed genes (Table 2). Fifteen of these pathways are predicted to be activated in the intestine of insulin-resistant subjects, and almost half of them are known to participate in inflammatory cell signaling pathways (JNK, TREM1, IL1α, IFNγ, SELPLG, P38 MAPK and TLR4). Interestingly, the IPA analysis predicts the activation of the p38 MAPK pathways, which is coherent with our previous study [10]. Indeed, we have demonstrated that p38 MAPK activating phosphorylation is increased in the intestine of insulin-resistant subjects compared to the intestine of insulin-sensitive subjects [10]. Other pathways found to be upregulated were involved in mitosis (PDGF, ERK and MAP2K1/2), transcription (C/EBPα, NUPR1, SMAD4), immunity (CD40LG) and coagulation (F7). Concerning the two pathways that are predicted to be down-regulated in the intestine of insulin-resistant subjects, we noticed that immunity (immunoglobulin G, IgG) and gene expression regulation (histone deacetylases, HDAC) seem to be disturbed in presence of insulin resistance. Results of this analysis reveal several changes in the activation of key metabolic pathway and clearly suggest that insulin resistance is involved in the development of a chronic low grade pro-inflammatory intestinal microenvironment.

**Table 2:** Biological processes significantly enriched in differentially expressed genes in the intestine of insulin-resistant subjects

Regulators	Role	State (z-score)	*p*	Target genes regulated
PDGF	Mitogenic	↗ (3.41)	10^−16^	ATF3, CNN1, CTGF, CYR61, DUSP1, EGR1, FOS, FOSB, GEM, IL1B, JUN, KLF6, RGS1, SLC2A3, THBS1, ZFP36
Jnk	Inflammation	↗ (2.77)	10^−07^	BIRC3, CTGF, DUSP1, IL1B, JUN, PTGS2, VCAM1, ZFP36
ERK	Mitogenic	↗ (2.62)	10^−09^	CTGF, DUSP1, EGR1, FOS, HBEGF, JUN, MUC5AC, PDK4, PTGS2, THBS1, ZFP36
F7	Coagulation	↗ (2.40)	10^−07^	CTGF, EGR1, FOS, HBEGF, IL1B, ZFP36
TREM1	Inflammation	↗ (2.33)	10^−05^	ATF3, CFB, EGR1, GADD45B, GEM, HBEGF, IL1B, PTGS2, RGS1, THBS1
C/EBPα	Transcription	↗ (2.22)	10^−03^	BTG2, CSF3R, CXCR4, PTGS2, S100A9
CD40LG	Immunity	↗ (2.20)	10^−05^	BIRC3, E2F2, HLA-DQA1, HLA-DRB4, IL1B, PTGS2, VCAM1
MAP2K1/2	Mitogenic	↗ (2.19)	10^−05^	ATF3, CTGF, DUSP1, EGR1, IL1B
IL1α	Inflammation	↗ (2.17)	10^−03^	BIRC3, IL1B, PTGS2, S100A9, SERPINA3
IFNγ	Inflammation	↗ (2.16)	10^−03^	ATF3, CXCR4, HLA-A/C/DQB1, IL1B, KLF6, PTGS2, VCAM1
NUPR1	Transcription	↗ (2.12)	10^−01^	ATF3, BRI3BP, CXCR4, CYR61, HBEGF, KLF6, SIK1, ZFAND2
SELPLG	Inflammation	↗ (2.00)	10^−04^	CLK1, CXCR4, FOS, IL1B
SMAD4	Transcription	↗ (2.00)	10^−02^	CTGF, GADD45B, IRAK3, THBS1
P38 MAPK	Inflammation	↗ (2.00)	10^−06^	ATF3, CD55, CTGF, EGR1, FOS, IL1B, JUN, MUC5AC, PTGS2
TLR4	Inflammation	↗ (2.00)	10^−05^	ATF3, IL1B, IRAK3, MUC5AC, VCAM1
IgG	Immunity	↘ (−2.24)	10^−03^	DUSP1, IL1B, LDLR, SLC2A3, ZFP36
Hdac	Deacetylases	↘ (−2.57)	10^−07^	ATF3, EGR1, FOS, GADD45B, JUN, KLF6, KLF9

↗: Biological processes prediced to be increased

↘: Biological processes predicted to be decreased

The most significant Gene Ontology (GO) terms identified by GO enrichment analysis, listed in the [see Additional file 1: Table S2], strongly support the results obtained with IPA. Indeed, several GO terms are related to: (i) immunity (GO: 0002682, 0006955, 0002376, 0050776, 0002684); (ii) digestion process (GO:0007586, 0008152, 0044248, 0002684); and (iii) response to oxidative stress and inflammation (GO:0006950, 1901700, 0033554, 0009991). This analysis has also identified novel cellular processes that are possibly altered in the intestine of insulin-resistant subjects. GO terms concerning cell death regulation were particularly frequent in the top list of GO biological processes (GO: 0010941, 0042981, 0043067, 0042981, 0043068, 0043065). Finally, several GO terms involved in cellular response to a broad-ranging number of stimulus (GO: 0009991, 0050896, 0010035, 0008152, 0010033, 0009605, 0051716, 0009725, 0009719, 0031667) as well as in several general metabolic process (GO: 0008152, 0044238, 0071704, 0044281, 0006629, 0008202) are significantly enriched in insulin-resistant subjects, thereby providing an additional knowledge related to the impact of insulin resistance on enterocyte metabolism.

**Fig. 2:**
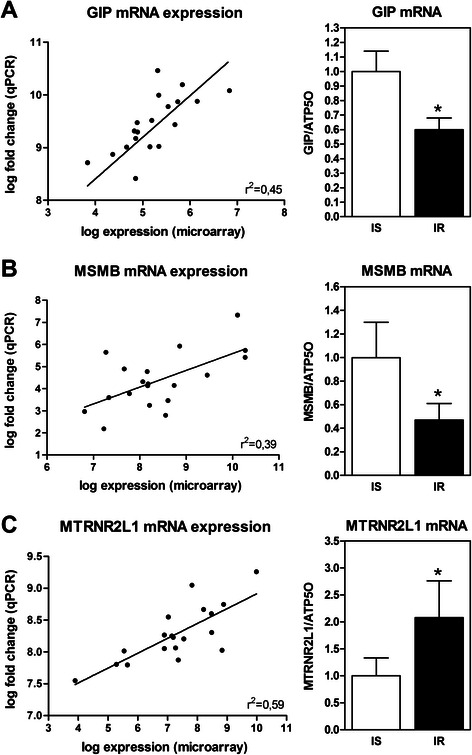
Differentially expressed genes in the intestine of insulin-resistant compared to insulin-sensitive subjects. *Left panel* illustrate spearman correlation of gene expression levels obtained with microarray and mRNA expression measure by qPCR. *Right panel* illustrate mRNA expression measured by qPCR of **a** *GIP*, **b** *MSMB*, and **c** *MTRNR2L1* in the intestine of insulin-sensitive (IS) (*n* = 9) and insulin-resistant (IR) subjects (*n* = 9). * *p* < 0.05 (Wilcoxson signed-rank test)

Using more severe parameter analysis, three genes are significantly and differently expressed in the intestine of insulin-resistant subjects compared to insulin-sensitive controls (fold change > 1.5 and FDR < 0.05). Insulinotropic glucose-dependent peptide (*GIP*, 9.85 ± 0.36 vs. 9.06 ± 0.37) and β-microseminoprotein (*MSMB*, 8.79 ± 1.16 vs. 7.54 ± 0.68) transcripts are significantly reduced in the intestine of insulin-resistant subjects compared to the intestine of insulin-sensitive controls. Inversely, expression levels of the *MTRNR2L1* gene, coding for the humanin like-1 protein, are significantly higher (7.99 ± 0.39 vs. 8.58 ± 0.24) in the intestine of insulin-resistant subjects than of insulin-sensitive controls. Changes in expression levels of these 3 genes are successfully replicated using qPCR as shown in the Fig. 2, right panel. qPCR fold changes of each target gene significantly correlate with the expression values obtained in the microarray experiments (Fig. 2, left panel). This validation confirms that gene expression levels of *GIP* and *MSMB* is reduced by 40 % (range 30–49 %) and 53 % (range 32–67 %), respectively, but the *MTRNR2L1* mRNA levels are increased by 193 % (range 72–401 %) in the intestine of insulin-resistant obese subjects compared to the intestine of insulin-sensitive obese subjects.

Linear regression analysis is performed to explore the association of anthropometric and biochemical parameters with mRNA expression of aforementioned genes. As expected, HOMA-IR index is a significant correlate of *GIP*, *MSMB* and *MTRNR2L1* mRNA expression (*r* = ±0.50–0.61, *n* = 18, *p* < 0.01). While *GIP* and *MSMB* mRNA levels are not associated with BMI, *MTRNR2L1* mRNA expression tends to be positively associated with this parameter (*r* = 0.43, *n* = 18, *p* = 0.6). High fasting levels of TG, which is a common component of insulin resistance, are significantly associated with *GIP* (*r* = −0.43, *n* = 18, *p* = 0.05) and *MTRNR2L1* (*r* = 0.65, *n* = 18, *p* < 0.01) but only tend to be associated with MSMB (*r* = −0.40, *n* = 18, *p* = 0.08). Sex and age are not associated with the expression of these genes. Our findings suggest that *GIP* and *MSMB* expression levels are reduced in the intestine in presence of insulin resistance and dyslipidemia while increased *MTRNR2L1* intestinal expression seems to be a function of alterations in increased adiposity and the presence of glucose homeostasis and lipid metabolism.

**Fig. 3:**
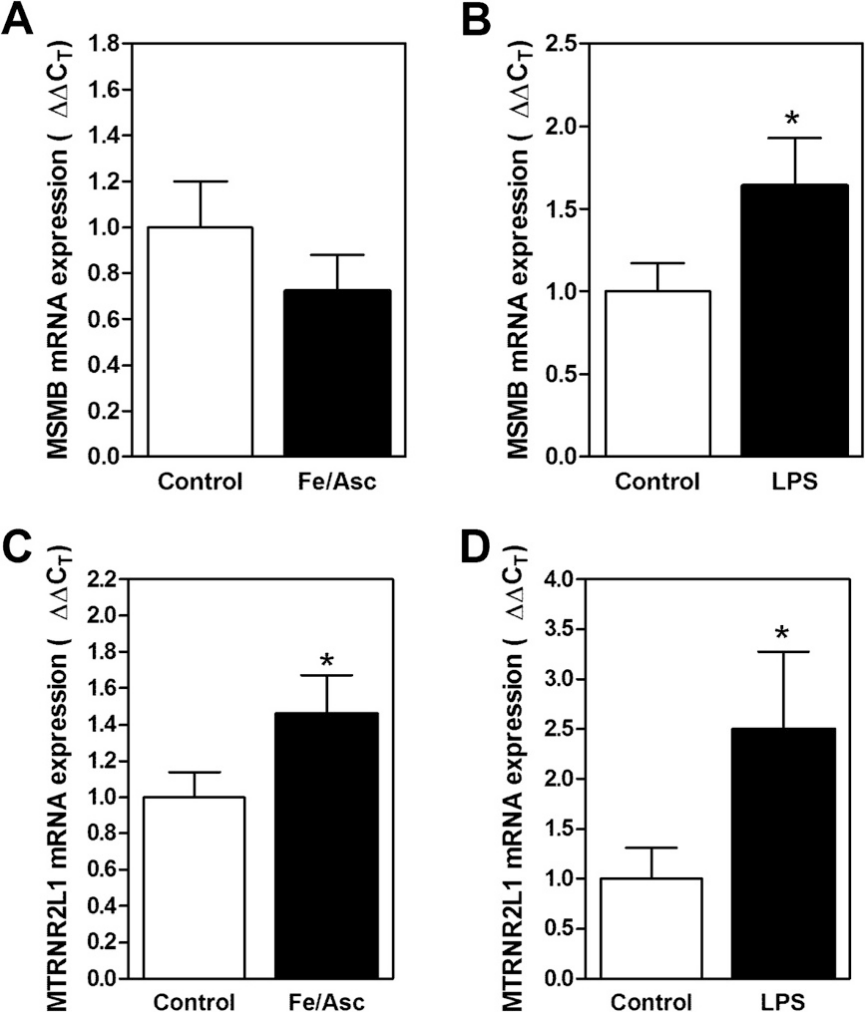
**a**-**b** *MSMB*, and **c**-**d** *MTRNR2L1* mRNA expression levels in Caco-2/15 cells following exposure to oxidative stress and inflammatory conditions. Caco-2/15 cells were cultured in the presence or absence of (**a-c**) Fe/Asc (200 μM/2 mM) for 6 h and of (**b**-**d**) LPS (150 μg/mL) for 24 h. Messenger RNA expression was measured by qPCR (ΔΔCt). * *p* < 0.05 (Student’s *t*-test)

As intestinal insulin resistance seems to be triggered by exacerbated oxidative stress and low-grade inflammation, we have tested the impact of iron-ascorbate (Fe/Asc) oxygen radical-generating system [16, 21] and lipopolysaccharide (LPS)-mediated inflammation [16] on the mRNA expression status of *MSMB* and *MTRNR2L1* in Caco-2/15 cells. Since *GIP* has been found expressed only in enteroendocrine K-cells, and mRNA levels of this gene have not been assessed in enterocytes. On the other hand, MSMB and MTRNR2L1 were previously demonstrated to be expressed in various epithelial cells [22, 23]. Treatment of Caco-2/15 cells with Fe/Asc do not significantly alter *MSMB* mRNA levels while LPS administration induce it (Fig. 3a, b). The addition of Fe/Asc and LPS to the cell culture medium significantly increases the mRNA expression of *MTRNR2L1* (Fig. 3c, d). Therefore, cellular *MTRNR2L1* displays the same expression pattern observed in the intestine of insulin-resistant subjects, which is not exactly the case for *MSMB*.

## Discussion

Insulin signaling defects can disturb metabolic functions in liver, adipose tissue and muscle. They represent key pharmacological targets to alleviate insulin resistance-related risk factors or diseases [24]. Animal and human studies have recently allowed to display a similar behavior in the intestine of obese and insulin-resistant subjects [3–10, 25, 26]. Since these defects were accompanied with an intestinal TRL overproduction, we have argued that postprandial dyslipidemia associated to insulin resistance and T2D is at least partially driven by an adverse intestinal lipid metabolism [11, 27]. In fact, we could test this hypothesis in human intestine, which allowed us to show that defects in insulin signalling homeostasis are related to the presence of oxidative stress and chronic low-grade inflammation [10]. The aim of the present investigation is to extend these findings by identifying additional intestinal key factors implicated in these disorders using the powerful RNA microarray approach using the intestine of individuals with a relatively low or high systemic insulin resistance state. We have detected 195 differentially expressed genes and have found that 36 of them were previously noted to be involved in specific intestinal functions, including digestion, extracellular matrix, endocrine system, immunity, inflammation/oxidative stress and cholesterol metabolism. Moreover, these differentially expressed genes point to a potential activation of several metabolic pathways associated with cell growth, immunity, transcription regulation and especially inflammatory cell signaling. Other cellular processes identified by GO analysis include cell death regulation, response to stimulus (i.e., nutrients, hormones, inflammation) and metabolic processes. Using more stringent analysis criteria (fold change > 1.5 and FDR < 0.05), we have discovered 3 differentially expressed genes in the intestine of insulin-resistant compared to the intestine of insulin-sensitive obese subjects. Intestinal transcripts of the *GIP* and *MSMB* are down-regulated, but those of *MTRNR2L1* show a significant increase in insulin-resistant subjects. These observations suggest that while mRNA profile seems relatively stable in the intestine of obese subjects, the expression of few specific genes are altered in conditions of insulin resistance. Interestingly, we observe that *MTRNR2L1* mRNA can be altered by either oxidative stress or inflammation in Caco-2/15 cells. This study contributes to the identification of potential key factors implicated in aberrant intestinal adaptation leading to increased postprandial lipid and lipoprotein synthesis in presence of systemic insulin resistance. This condition is known to contribute to atherogenic dyslipidemia characteristic to obese and T2D subjects.

As mentioned before, the transcript profiling indicated that some genes are differentially expressed in the intestine of insulin-resistant subjects compared to the intestine of insulin-sensitive subjects. A large number of these genes are known to play a key role in the intestine. Genes coding for enzymes involved in food digestion are particularly well represented on this list as well as in the top-ranked GO terms. Although no particular tendency is observed in the expression of these genes between the two groups of subjects, they are involved in the hydrolysis of all nutrient classes (i.e., carbohydrates, proteins and lipids). An altered expression pattern is also observed in some extracellular matrix-related genes, especially those coding for mucin proteins. The latter create an insoluble mucous barrier that serves to protect intestinal wall integrity and to coordinate the interactions between intestinal luminal content and epithelial cells [28]. Increased mucin mass as well as alterations in the expression profile of a number of mucin members were previously reported in diabetic animal models [29]. The association of *Akkermansia muciniphila,* a mucin-degrading bacteria residing in the mucus layer, in conditions of obesity and systemic insulin resistance, further supports the hypothesis that the mucin-dependent mucus layer plays a key role in intestinal dysregulation [30]. In addition to the observed reduction of the *GIP* expression in presence of insulin resistance, three other intestinal peptides are differentially regulated. Gastrokine 1 and 2 are down-regulated in the intestine of insulin-resistant subjects, an observation in line with previous studies reporting decreased gastrokine expression in pro-inflammatory environment [31]. On the other hand, the increased levels of vasoactive intestinal polypeptide (*VIP*) transcript in the intestine of insulin-resistant subjects corroborates previous observation of higher VIP levels in obese, dyslipidemic and insulin-resistant subjects [32].

The presence of elevated oxidative stress and low-grade inflammation has been hypothesized to be intimately related to progression of insulin resistance and other obesity-related disorders [33]. We have previously examined several oxidative stress and inflammatory markers in the intestine of severely obese subjects with insulin resistance [10]. Reduced antioxidant defence and higher levels of lipid peroxidation by-product are observed in these subjects. Moreover, increased activation of inflammatory pathways (i.e., p38 MAPK and NF-κB) in presence of insulin resistance is evidenced together with elevated protein mass of IL-6, ICAM and COX-2, as well as of the mRNA levels of TNF-α, [10]. The present findings provide additional evidence that systemic insulin resistance is associated with the induction of several intestinal inflammatory pathways. Indeed, eight differentially expressed genes related to inflammation and oxidative stress are identified in the intestine of insulin-resistant subjects using the microarray approach. Among them, it is worth mentioning COX-2 (the rate limiting enzyme of prostaglandin biogenesis) that showed higher mRNA expression levels in the intestine of insulin-resistant subjects. This observation is coherent with our previous observations related to increased COX-2 protein levels in these subjects [10]. Several top-ranked significantly enriched GO terms are related to stress response and inflammation. More specifically, analysis of the present data have identified several inflammatory pathways enriched in differentially expressed genes, including Jnk, TREM1, IL1α, IFNγ, SELPLG, p38-MAPK and TLR4. Interestingly, p38-MAPK pathway activation in presence of systemic insulin-resistance has also been previously confirmed by our group in the intestine of these subjects, as monitored by the phosphorylation state of the p38 MAPK kinase [10]. Using the two approaches, i.e., GO enrichment analysis and IPA, we have found that insulin-resistance state is also associated with gene profiling remodeling related to immunity [i.e., several members of the class I and II major histocompatibility complex (MHC)]. Alterations in the expression of these genes have been previously demonstrated in the adipocytes of obese subjects [34]. Moreover, mice deficient in class II MHC develop less inflammation in the adipose tissue and remained insulin sensitive when fed with a high-fat diet, an effect apparently mediated by macrophage and T-cell activation [34]. Apparently, changes in the expression profile of MHC genes may be associated with the exacerbated inflammatory state and decreased insulin response in the small intestine of insulin-resistant subjects, but additional work is necessary to determine the mechanisms. Collectively, these data support the hypothesis that oxidative stress and chronic low-grade inflammation are exacerbated in the intestine of insulin-resistant subjects, which may contribute to reducing local insulin sensitivity while promoting intestinal dysregulation leading to postprandial dyslipidemia. Finally, the GO enrichment analysis has deepened the analysis of the data through the identification of three novel families linked to cellular processes and modulated by the presence of systemic insulin resistance. The presence of several GO terms concerning cell death regulation in the top-ranked GO processes suggest that an abnormal regulation of the small intestine epithelium renewal may be involved in this condition. GO terms related to the cellular response to a broad-ranging number of stimulus as well as in several general metabolic processes are also significantly enriched in insulin-resistant subjects. These latter GO terms suggest that insulin-resistance profoundly alter enterocyte metabolic response to stimulus from it immediate environment, which supports the hypothesis of dysfunctional enterocytes leading to postprandial dyslipidemia in insulin-resistant subjects.

A robust and interesting finding of this study is the identification of three differentially expressed genes in the intestine of insulin-resistant obese subjects. Messenger RNA changes of *GIP*, *MSMB* and *MTRNR2L1* genes have been validated by qPCR in the same intestinal samples. GIP, one of the two major incretin hormones with the glucagon-like peptide (GLP1), is secreted by enteroendocrine K-cells in the proximal small intestine in response to glucose hyperosmolality. This gut hormone amplifies pancreatic insulin secretion and stimulates proliferation of pancreatic β-cells. Besides the pancreas, the GIP receptor is also expressed in adipose tissue, brain and stomach as well as in the proximal portion of the small intestine [35]. In this context, GIP was found to stimulate dipeptide and glucose absorption in enterocytes, and to moderately inhibit gastric acid secretion and gastric emptying [36]. Interestingly, the decreased GIP expression levels noted in the present work is coherent with previous studies on insulin resistance and T2D [35], reporting reduced β-cell responsiveness to GIP [37] and lower postprandial GIP circulating levels in diabetes [38]. We may therefore deduce that *GIP* mRNA reduction in the intestine exposed to systemic insulin resistance corroborates GIP signaling defects in insulin-resistant and T2D subjects. Down-regulation of GIP expression may suggest reduced mRNA expression levels or may alternatively indicate a decrease in the number of K-cells in the duodenum in presence of systemic insulin resistance. While the precise GIP functions, especially in non-pancreatic tissue, remained to be explored, the decreased GIP expression in the intestine has the potential to influence overall energy metabolism through the regulation of insulin secretion.

Messenger RNA of the *MTRNR2L1* gene, coding for humanin-like 1 protein, is increased in the intestine of insulin-resistant subjects compared to the intestine of insulin-sensitive subjects. This gene is one of the numerous nuclear copies of the mitochondrial *MTRNR2* gene coding for the humanin protein [22]. While little is known on these nuclear copies, some authors have reported that mitochondrial humanin is linked to insulin resistance [39]. We have noticed that the expression of the two genes, *MTRNR2L1* and *MTRNR2,* is upregulated in the intestine of subjects with insulin resistance (Fig. 2c and data not shown), which supports the possibility that both genes maintain a similar expression pattern during evolution [22]. This gene family codes for highly similar small secretory 21 to 24- amino acid peptides [22]. While they were initially identified as survival factors in the context of Alzheimer disease [40], they also seem to protect cells from oxidative stress, starvation and hypoxia [41]. Increased humanin protein mass was noted in atherosclerotic plaques of symptomatic subjects and in epithelial cells of *ApoE*-deficient mice on high-cholesterol diet to possibly protect cells against apoptosis [42]. Accordingly, we could successfully demonstrate that pro-oxidant (i.e., Fe/Asc) and pro-inflammatory (i.e., LPS) stimuli induce *MTRNR2L1* mRNA expression in Caco-2/15 cells. Potential humanin-like 1 role in cell may therefore represent an attempt to alleviate adverse effects of oxidative stress on enterocytes. However, further studies are needed to define the precise regulation and function of humanin and humanin-like proteins in this tissue.

We also observe a decreased expression of the β-microseminoprotein, a member of the immunoglobulin binding factor family. This protein is expressed in several epithelial cells and has been shown to inhibit cancer cell growth and to possess antifungal activity [23]. There is presently no available information reporting a potential link between metabolic diseases and the β-microseminoprotein. Moreover, a significant role in small intestine cancer prevalence is improbable as it remains a very uncommon cancer even in obese subjects [43].

This study represents a unique opportunity to assess the impact of systemic insulin resistance on intestinal metabolism, but we acknowledge some limitations. Due to the technical difficulty inherent to intestinal sampling, only subjects with obesity were included in his study and some observations could be specific to this population. The cross-sectional design prevents us to infer about a cause-effect relationship between intestinal gene expression and intestinal metabolic dysregulation or dyslipidemia in these subjects. Specific experiments and longitudinal studies should be performed to definitively conclude on the involvement of these genes in the establishment of intestinal insulin resistance and postprandial lipid metabolism alterations.

## Conclusion

We have demonstrated that human small intestine transcriptome show evidences of functional adaptations when exposed to systemic insulin resistance. Genes involved in digestion, extracellular matrix, intestinal peptides and cholesterol metabolism were differentially regulated in the intestine of insulin-resistant subjects compared to the intestine of insulin-sensitive subjects. Nevertheless, insulin resistance was particularly associated with an amplification of oxidative stress and chronic low-grade inflammation which should now be considered as potential contributors to the development of an altered intestinal lipid metabolism in an unfavorable environment. Some genes and pathways identified are likely to contribute to the development of a dysfunctional enteric state leading to lipid and lipoprotein metabolic alterations and to the postprandial dyslipidemia commonly observed in patients with insulin resistance and T2D. Additional studies are needed to define whether these changes are related to the progression of intestinal metabolism dysregulation or represent an adaptive response to this state.

## Additional file

Additional file 1 **Table S1.** Complete list of the 195 annotated differentially expressed genes in the intestine of insulin-resistant compared to insulin-sensitive subjects (Fold change > 1.2, P < 0.05). **Table S2.** List of the GO terms significantly enriched in differentially expressed genes in the intestine of insulin-resistant compared to insulin-sensitive subjects (FDR < 10-5). (PDF 122 kb)

## References

[1] Duez H, Lamarche B, Uffelman KD, Valero R, Cohn JS, Lewis GF (2006). Hyperinsulinemia is associated with increased production rate of intestinal apolipoprotein B-48-containing lipoproteins in humans. Arterioscler Thromb Vasc Biol.

[2] Marcil V, Peretti N, Delvin E, Levy E (2004). Digestive and absorptive processes of lipids. Gastroentérologie Clin Biol.

[3] Haidari M, Leung N, Mahbub F, Uffelman KD, Kohen-Avramoglu R, Lewis GF, Adeli K (2002). Fasting and postprandial overproduction of intestinally derived lipoproteins in an animal model of insulin resistance. Evidence that chronic fructose feeding in the hamster is accompanied by enhanced intestinal de novo lipogenesis and ApoB48-containing lipoprotein overproduction. J Biol Chem.

[4] Vine DF, Takechi R, Russell JC, Proctor SD (2007). Impaired postprandial apolipoprotein-B48 metabolism in the obese, insulin-resistant JCR:LA-cp rat: increased atherogenicity for the metabolic syndrome. Atherosclerosis.

[5] Zoltowska M, Ziv E, Delvin E, Sinnett D, Kalman R, Garofalo C, Seidman E, Levy E (2003). Cellular aspects of intestinal lipoprotein assembly in psammomys obesus a model of insulin resistance and type 2. Diabetes.

[6] Adeli K, Lewis GF (2008). Intestinal lipoprotein overproduction in insulin-resistant states. Curr Opin Lipidol.

[7] Lewis GF, Naples M, Uffelman K, Leung N, Szeto L, Adeli K (2004). Intestinal lipoprotein production is stimulated by an acute elevation of plasma free fatty acids in the fasting state: studies in insulin-resistant and insulin-sensitized Syrian golden hamsters. Endocrinology.

[8] Lewis GF, Uffelman K, Naples M, Szeto L, Haidari M, Adeli K (2005). Intestinal lipoprotein overproduction, a newly recognized component of insulin resistance, is ameliorated by the insulin sensitizer rosiglitazone: studies in the fructose-fed Syrian golden hamster. Endocrinology.

[9] Federico LM, Naples M, Taylor D, Adeli K (2006). Intestinal insulin resistance and aberrant production of apolipoprotein B48 lipoproteins in an animal model of insulin resistance and metabolic dyslipidemia evidence for activation of protein tyrosine phosphatase-1B, extracellular signal–related kinase, and sterol regulatory element–binding protein-1c in the fructose-Fed hamster intestine. Diabetes.

[10] Veilleux A, Grenier E, Marceau P, Carpentier AC, Richard D, Levy E (2014). Intestinal lipid handling: evidence and implication of insulin signaling abnormalities in human obese subjects. Arterioscler Thromb Vasc Biol.

[11] Couture P, Tremblay AJ, Kelly I, Lemelin V, Droit A, Lamarche B (2014). Key intestinal genes involved in lipoprotein metabolism are downregulated in dyslipidemic men with insulin resistance. J Lipid Res.

[12] Phillips C, Mullan K, Owens D, Tomkin GH (2006). Intestinal microsomal triglyceride transfer protein in type 2 diabetic and non-diabetic subjects: the relationship to triglyceride-rich postprandial lipoprotein composition. Atherosclerosis.

[13] McNamara JR, Shah PK, Nakajima K, Cupples LA, Wilson PW, Ordovas JM, Schaefer EJ (2001). Remnant-like particle (RLP) cholesterol is an independent cardiovascular disease risk factor in women: results from the Framingham Heart Study. Atherosclerosis.

[14] Matthews DR, Hosker JP, Rudenski AS, Naylor BA, Treacher DF, Turner RC (1985). Homeostasis model assessment: insulin resistance and beta-cell function from fasting plasma glucose and insulin concentrations in man. Diabetologia.

[15] Beaulieu JF, Quaroni A (1991). Clonal analysis of sucrase-isomaltase expression in the human colon adenocarcinoma Caco-2 cells. Biochem J.

[16] Denis MC, Furtos A, Dudonné S, Montoudis A, Garofalo C, Desjardins Y, Delvin E, Levy E (2013). Apple peel polyphenols and their beneficial actions on oxidative stress and inflammation. PLoS One.

[17] Irizarry RA, Hobbs B, Collin F, Beazer-Barclay YD, Antonellis KJ, Scherf U, Speed TP (2003). Exploration, normalization, and summaries of high density oligonucleotide array probe level data. Biostat Oxf Engl.

[18] Tusher VG, Tibshirani R, Chu G (2001). Significance analysis of microarrays applied to the ionizing radiation response. Proc Natl Acad Sci U S A.

[19] Whyte L, Huang Y-Y, Torres K, Mehta RG (2007). Molecular mechanisms of resveratrol action in lung cancer cells using dual protein and microarray analyses. Cancer Res.

[20] Smith LK, Tandon A, Shah RR, Mav D, Scoltock AB, Cidlowski JA (2013). Deep sequencing identification of novel glucocorticoid-responsive miRNAs in apoptotic primary lymphocytes. PLoS ONE.

[21] Courtois F, Seidman EG, Delvin E, Asselin C, Bernotti S, Ledoux M, Levy E (2003). Membrane peroxidation by lipopolysaccharide and iron-ascorbate adversely affects Caco-2 cell function: beneficial role of butyric acid. Am J Clin Nutr.

[22] Bodzioch M, Lapicka-Bodzioch K, Zapala B, Kamysz W, Kiec-Wilk B, Dembinska-Kiec A (2009). Evidence for potential functionality of nuclearly-encoded humanin isoforms. Genomics.

[23] Sutcliffe S, De Marzo AM, Sfanos KS, Laurence M (2014). MSMB variation and prostate cancer risk: clues towards a possible fungal etiology. Prostate.

[24] Filz HP (2000). Insulin sensitizer. A new therapy option for type 2 diabetic patients]. MMW Fortschr Med.

[25] Levy E, Spahis S, Ziv E, Marette A, Elchebly M, Lambert M, Delvin E (2006). Overproduction of intestinal lipoprotein containing apolipoprotein B-48 in Psammomys obesus: impact of dietary n-3 fatty acids. Diabetologia.

[26] Qin B, Qiu W, Avramoglu RK, Adeli K (2007). Tumor necrosis factor-α induces intestinal insulin resistance and stimulates the overproduction of intestinal apolipoprotein B48-containing lipoproteins. Diabetes.

[27] Duez H, Lamarche B, Valéro R, Pavlic M, Proctor S, Xiao C, Szeto L, Patterson BW, Lewis GF (2008). Both intestinal and hepatic lipoprotein production are stimulated by an acute elevation of plasma free fatty acids in humans. Circulation.

[28] Johansson MEV, Sjövall H, Hansson GC (2013). The gastrointestinal mucus system in health and disease. Nat Rev Gastroenterol Hepatol.

[29] Daft J, Lorenz R (2014). Antimicrobial peptide and mucin expression differ between the diabetic NOD mouse and non-diabetic NOR mouse (650.16). FASEB J.

[30] Anhe FF, Roy D, Pilon G, Dudonne S, Matamoros S, Varin TV, Garofalo C, Moine Q, Desjardins Y, Levy E, Marette A (2015). A polyphenol-rich cranberry extract protects from diet-induced obesity, insulin resistance and intestinal inflammation in association with increased Akkermansia spp. population in the gut microbiota of mice. Gut.

[31] Baus-Loncar M, Lubka M, Pusch CM, Otto WR, Poulsom R, Blin N (2007). Cytokine regulation of the trefoil factor family binding protein GKN2 (GDDR/TFIZ1/blottin) in human gastrointestinal epithelial cells. Cell Physiol Biochem Int J Exp Cell Physiol Biochem Pharmacol.

[32] Tomkin GH, Ardill J, Lafferty H, Darragh A (1983). Vasoactive intestinal polypeptide in obesity. Int J Obes.

[33] Elmarakby AA, Sullivan JC (2012). Relationship between oxidative stress and inflammatory cytokines in diabetic nephropathy. Cardiovasc Ther.

[34] Deng T, Lyon CJ, Minze LJ, Lin J, Zou J, Liu JZ, Ren Y, Yin Z, Hamilton DJ, Reardon PR, Sherman V, Wang HY, Phillips KJ, Webb P, Wong STC, Wang R-F, Hsueh WA (2013). Class II major histocompatibility complex plays an essential role in obesity-induced adipose inflammation. Cell Metab.

[35] Ahrén B (2013). Incretin dysfunction in type 2 diabetes: clinical impact and future perspectives. Diabetes Metab.

[36] Coon SD, Schwartz JH, Rajendran VM, Jepeal L, Singh SK (2013). Glucose-dependent insulinotropic polypeptide regulates dipeptide absorption in mouse jejunum. Am J Physiol Gastrointest Liver Physiol.

[37] Belinova L, Kahleova H, Malinska H, Topolcan O, Vrzalova J, Oliyarnyk O, Kazdova L, Hill M, Pelikanova T (2014). Differential acute postprandial effects of processed meat and isocaloric vegan meals on the gastrointestinal hormone response in subjects suffering from type 2 diabetes and healthy controls: a randomized crossover study. PLoS One.

[38] Vilsbøll T, Krarup T, Deacon CF, Madsbad S, Holst JJ (2001). Reduced postprandial concentrations of intact biologically active glucagon-like peptide 1 in type 2 diabetic patients. Diabetes.

[39] Kuliawat R, Klein L, Gong Z, Nicoletta-Gentile M, Nemkal A, Cui L, Bastie C, Su K, Huffman D, Surana M, Barzilai N, Fleischer N, Muzumdar R (2013). Potent humanin analog increases glucose-stimulated insulin secretion through enhanced metabolism in the β cell. FASEB J Off Publ Fed Am Soc Exp Biol.

[40] Hashimoto Y, Niikura T, Ito Y, Sudo H, Hata M, Arakawa E, Abe Y, Kita Y, Nishimoto I (2001). Detailed characterization of neuroprotection by a rescue factor humanin against various Alzheimer’s disease-relevant insults. J Neurosci Off J Soc Neurosci.

[41] Yen K, Lee C, Mehta H, Cohen P (2013). The emerging role of the mitochondrial-derived peptide humanin in stress resistance. J Mol Endocrinol.

[42] Zacharias DG, Kim SG, Massat AE, Bachar AR, Oh YK, Herrmann J, Rodriguez-Porcel M, Cohen P, Lerman LO, Lerman A (2012). Humanin, a cytoprotective peptide, is expressed in carotid atherosclerotic [corrected] plaques in humans. PLoS One.

[43] Hall EH, Crowe SE (2011). Environmental and lifestyle influences on disorders of the large and small intestine: implications for treatment. Dig Dis Basel Switz.

