# Supplementary material for: Altered intestinal functions and increased local inflammation in insulin-resistant obese subjects: a gene-expression profile analysis
Source: BMC Gastroenterol. 2015 Sep 16;15:119. doi: 10.1186/s12876-015-0342-y (PMC4574092; doi:10.1186/s12876-015-0342-y)
Supplement: Additional file 1 — Table S1. Complete list of the 195 annotated differentially expressed genes in the intestine of insulin-resistant compared to insulin-sensitive subjects (Fold change > 1.2, P < 0.05). Table S2. List of the GO terms significantly enriched in differentially expressed genes in the intestine of insulin-resistant compared to insulin-sensitive subjects (FDR < 10-5). (PDF 122 kb) [file 12876_2015_342_MOESM1_ESM.pdf]

Supplementary Table 1: Complete list of the 195 annotated differentially expressed genes in the intestine of insulin-resistant compared to insulin-sensitive subjects (Fold change > 1.2, P < 0.05).

Symbol	Fold Change	Illumina	Entrez Gene ID
ACAA1	-1,233	ILMN_1738921	30
ACSL5	-1,225	ILMN_2370882	51703
ACY1	-1,228	ILMN_1683883	95
ADA	-1,218	ILMN_1803686	100
ADAMDEC1	1,224	ILMN_1716909	27299
ADH4	-1,211	ILMN_2136010	127
ADTRP	1,218	ILMN_2078592	84830
AGO2	-1,243	ILMN_1695719	27161
AGPAT9	-1,332	ILMN_1794875	84803
AGR2	1,278	ILMN_1814151	10551
ALAS2	1,303	ILMN_2367126	212
ALDH3A1	-1,213	ILMN_1702503	218
AOC1	-1,379	ILMN_1731433	26
APOA1	-1,373	ILMN_1690884	335
APOD	1,203	ILMN_1780170	347
AQP11	-1,278	ILMN_1762606	282679
AQP2	-1,210	ILMN_1732149	359
ARL4A	1,229	ILMN_1775405	10124
ARPC4	1,244	ILMN_2393763	10093
ATF3	1,402	ILMN_2374865	467
BIRC3	1,202	ILMN_1776181	330
BRI3BP	-1,226	ILMN_1797693	140707
BTG2	1,256	ILMN_1770085	7832
C15orf48	-1,391	ILMN_1805410	84419
C8orf4	1,302	ILMN_1656369	56892
CALD1	-1,224	ILMN_1717990	800
CASP7	-1,206	ILMN_2373763	840
CCBE1	-1,205	ILMN_2122511	147372
CD55	1,213	ILMN_1800540	1604
CD79B	1,203	ILMN_2366212	974
CDH17	-1,221	ILMN_1814015	1015
CDHR2	-1,367	ILMN_1718173	54825
CDK11A/B	-1,316	ILMN_2330552	728642;984
CEACAM5	-1,243	ILMN_1670959	1048
CEACAM6	-1,291	ILMN_1712522	4680
CELA3A	1,223	ILMN_1677559	10136
CES2	-1,297	ILMN_2362681	8824
CFB	-1,204	ILMN_1774287	629
CLCA1	-1,250	ILMN_1797219	1179
CLDN18	-1,279	ILMN_1696284	51208

CLK1	1,230	ILMN_1679727	1195
CNN1	1,257	ILMN_1810054	1264
COL17A1	-1,250	ILMN_1651282	1308
COX7A1	1,212	ILMN_1662419	1346
CREB1	-1,225	ILMN_2334242	1385
CREB3L3	-1,320	ILMN_1779524	84699
CRIP1	-1,329	ILMN_1656920	1396
CSF3R	1,246	ILMN_2371280	1441
CSRNP1	1,251	ILMN_1703123	64651
CSRP2	1,244	ILMN_1660806	1466
CTGF	1,338	ILMN_2115125	1490
CXCR4	1,311	ILMN_1801584	7852
CYP2S1	-1,214	ILMN_1705403	29785
CYR61	1,252	ILMN_2188264	3491
DCN	1,238	ILMN_1768227	1634
DUSP1	1,366	ILMN_1781285	1843
E2F2	-1,270	ILMN_1777233	1870
EDN3	-1,210	ILMN_2358886	1908
EGR1	1,358	ILMN_1762899	1958
ERH	1,227	ILMN_1781795	2079
FABP2	-1,258	ILMN_1740479	2169
FAM107A	1,234	ILMN_1743445	11170
FAM118A	1,358	ILMN_1809147	55007
FBXO2	-1,213	ILMN_1803211	26232
FBXO6	-1,222	ILMN_1701455	26270
FCGBP	-1,303	ILMN_1718984	8857
FKBP5	1,385	ILMN_1778444	2289
FOS	1,546	ILMN_1669523	2353
FOSB	1,429	ILMN_1751607	2354
FXR1	1,219	ILMN_1679640	8087
GADD45B	1,262	ILMN_1718977	4616
GC	1,455	ILMN_1736162	2638
GEM	1,255	ILMN_2367883	2669
GIP	-1,723	ILMN_1673805	2695
GNL3L	-1,356	ILMN_1708414	54552
GOLM1	1,232	ILMN_1737011	51280
GP2	1,382	ILMN_2311873	2813
GPX8	1,206	ILMN_1767665	493869
GSTT1	1,832	ILMN_1730054	2952
GZMA	-1,275	ILMN_1779324	3001
H1F0	1,232	ILMN_1757467	3005
HBA1/2	1,932	ILMN_3240144	3040;3039
HBB	1,668	ILMN_2100437	3043
HBEGF	1,214	ILMN_2121408	1839
HDC	-1,222	ILMN_1792323	3067

HLA-A	2,631	ILMN_2165753	3105
HLA-C	-1,202	ILMN_1721113	3107
HLA-DQA1	1,378	ILMN_1808405	3117
HLA-DQB1	1,223	ILMN_1661266	3119
HLA-DRB1	-1,351	ILMN_1715169	3123
HLA-DRB4	1,391	ILMN_1752592	3126
HLA-F	-1,275	ILMN_1762861	3134
HMGCS1	1,240	ILMN_1797728	3157
HSH2D	-1,210	ILMN_1788017	84941
IFI35	-1,203	ILMN_1745374	3430
IL1B	1,212	ILMN_1775501	3553
INF2	-1,209	ILMN_2393450	64423
INSIG1	1,298	ILMN_1793474	3638
IRAK3	1,233	ILMN_1913678	11213
JUN	1,320	ILMN_1806023	3725
KLF6	1,202	ILMN_1735014	1316
KLF9	1,234	ILMN_1778523	687
KLRB1	1,211	ILMN_2079655	3820
LAMB3	-1,276	ILMN_1715684	3914
LCT	1,539	ILMN_2046073	3938
LDLR	1,295	ILMN_2053415	3949
LEAP2	-1,202	ILMN_2124361	116842
LRRC17	1,207	ILMN_1652826	10234
MAF	-1,287	ILMN_1719543	4094
MAPK13	-1,226	ILMN_1749327	5603
MED4	-1,236	ILMN_1664641	29079
MEG3	1,201	ILMN_2061435	55384
MEP1A	-1,260	ILMN_1659984	4224
MGP	1,218	ILMN_2071809	4256
MLN	-1,234	ILMN_1685858	4295
MMP7	1,273	ILMN_1685403	4316
MSMB	-1,803	ILMN_1699243	4477
MUC2	-1,321	ILMN_2205622	4583
MUC5AC	-1,575	ILMN_1748303	4586
MUC6	1,212	ILMN_1727875	4588
MVP	-1,244	ILMN_1803277	9961
MYL12A	-1,216	ILMN_1675848	10627
MYL9	1,217	ILMN_1776953	10398
NBPF10	-1,436	ILMN_2115490	55672
NEXN	1,213	ILMN_1783276	91624
NFIC	-1,202	ILMN_1675130	4782
NGFRAP1	1,243	ILMN_2370091	27018
NPIPB15	1,266	ILMN_2147105	728741
OPA3	-1,258	ILMN_2284591	80207
PARP12	-1,208	ILMN_1718558	64761

PCK2	-1,273	ILMN_1671791	5106
PDK4	1,314	ILMN_1684982	5166
PEBP1	-1,212	ILMN_1688089	5037
PGA5	-1,970	ILMN_1717572	643834
PJA2	1,361	ILMN_1688702	9867
PLIN2	-1,255	ILMN_1801077	123
PNLIPRP2	1,297	ILMN_1712613	5408
PRELID1	-1,238	ILMN_2179652	27166
PRKAR1A	1,202	ILMN_2389590	5573
PRSS1	1,236	ILMN_1734773	5644
PTGS2	1,202	ILMN_2054297	5743
PTK6	-1,213	ILMN_2075800	5753
RARRES2	1,232	ILMN_1810844	5919
RDH5	-1,206	ILMN_1773395	5959
REG1B	-1,537	ILMN_1681462	5968
REG4	-1,252	ILMN_2169383	83998
RERGL	1,281	ILMN_3243185	79785
RETSAT	-1,207	ILMN_1702633	54884
RGS1	1,252	ILMN_1656011	5996
RNASE1	1,339	ILMN_2333670	6035
RPL17	1,285	ILMN_1655422	6139
RPS23	1,262	ILMN_1772459	6228
RPS4Y1	1,266	ILMN_1783142	6192
S100A8	1,586	ILMN_1729801	6279
S100A9	1,510	ILMN_1750974	6280
S100P	-1,322	ILMN_1801216	6286
SCGB3A1	-1,286	ILMN_1679666	92304
SCIN	-1,227	ILMN_1813561	85477
SDCBP2	-1,253	ILMN_1705107	27111
SERPINA3	1,300	ILMN_1788874	12
SIK1	1,218	ILMN_3235647	150094
SLC22A18	-1,225	ILMN_2382505	5002
SLC28A2	-1,683	ILMN_1801886	9153
SLC2A3	1,254	ILMN_1775708	6515
SLC2A5	-1,249	ILMN_1671337	6518
SLC35G1	-1,221	ILMN_1690464	159371
SPARCL1	1,294	ILMN_1795251	8404
STMN2	1,209	ILMN_1795679	11075
SUSD2	-1,238	ILMN_1693270	56241
TFF1	-1,445	ILMN_1722489	7031
TFG	-1,227	ILMN_2341815	10342
THBS1	1,207	ILMN_1686116	7057
THNSL2	1,270	ILMN_2173294	55258
TIMM10	-1,255	ILMN_1765332	26519
TMEM176A	-1,227	ILMN_1791511	55365

TOP3A	-1,224	ILMN_2072973	7156
TRIM15	-1,365	ILMN_1754476	89870
TRIM31	-1,515	ILMN_1748685	11074
TRPV6	-1,233	ILMN_1674533	55503
TSC22D1	1,286	ILMN_1692177	8848
TSC22D3	1,285	ILMN_1748124	1831
UGT2B15	1,221	ILMN_2048414	7366
VAV3	-1,272	ILMN_2399463	10451
VCAM1	1,266	ILMN_2307903	7412
VIL1	-1,215	ILMN_1661750	7429
VIP	1,302	ILMN_2357542	7432
VRK3	-1,209	ILMN_1771697	51231
WDR72	1,306	ILMN_1763196	256764
WWP1	1,203	ILMN_1804328	11059
YY1	-1,254	ILMN_1770892	7528
ZAK	1,223	ILMN_1768110	51776
ZBTB16	1,232	ILMN_2305407	7704
ZFAND2A	-1,256	ILMN_1694671	90637
ZFP36	1,408	ILMN_1720829	7538
ZG16	-1,443	ILMN_2121774	653808

Supplementary Table 2: List of the GO terms significantly enriched in differentially expressed genes in the intestine of insulin-resistant compared to insulin-sensitive subjects (FDR < 10^{-5}).

GO terms	Description	P values	FDR q-value
0042221	response to chemical	$1,2x10^{-12}$	$1,7x10^{-08}$
0002682	regulation of immune system process	$4,4x10^{-12}$	$3,0x10^{-08}$
0007586	digestion	$7,9x10^{-12}$	$3,6x10^{-08}$
0006950	response to stress	$1,3x10^{-11}$	$4,5x10^{-08}$
0010941	regulation of cell death	$1,7x10^{-11}$	$4,8x10^{-08}$
0042981	regulation of apoptotic process	$3,4x10^{-11}$	$7,9x10^{-08}$
0043067	regulation of programmed cell death	$4,6x10^{-11}$	$8,9x10^{-08}$
0009991	response to extracellular stimulus	$8,0x10^{-11}$	$1,4x10^{-07}$
0008152	metabolic process	$9,7x10^{-11}$	$1,5x10^{-07}$
0010035	response to inorganic substance	$1,5x10^{-10}$	$2,1x10^{-07}$
0050896	response to stimulus	$3,1x10^{-10}$	$3,8x10^{-07}$
0044238	primary metabolic process	$4,8x10^{-10}$	$5,5x10^{-07}$
0010033	response to organic substance	$7,7x10^{-10}$	$8,1x10^{-07}$
0010942	positive regulation of cell death	$1,6x10^{-09}$	$1,6x10^{-06}$
0031667	response to nutrient levels	$2,1x10^{-09}$	$1,9x10^{-06}$
0009605	response to external stimulus	$2,4x10^{-09}$	$2,0x10^{-06}$
0071704	organic substance metabolic process	$4,0x10^{-09}$	$3,2x10^{-06}$
0002376	immune system process	$5,6x10^{-09}$	$4,2x10^{-06}$
0044710	single-organism metabolic process	$7,5x10^{-09}$	$5,4x10^{-06}$
0051716	cellular response to stimulus	$1,5x10^{-08}$	$9,9x10^{-06}$
0043068	positive regulation of programmed cell death	$1,6x10^{-08}$	$1,0x10^{-05}$
0044281	small molecule metabolic process	$1,8x10^{-08}$	$1,1x10^{-05}$
0002684	positive regulation of immune system process	$1,9x10^{-08}$	$1,1x10^{-05}$
0009725	response to hormone	$2,3x10^{-08}$	$1,3x10^{-05}$
0043065	positive regulation of apoptotic process	$2,3x10^{-08}$	$1,3x10^{-05}$
0009719	response to endogenous stimulus	$2,9x10^{-08}$	$1,5x10^{-05}$
1901700	response to oxygen-containing compound	$3,4x10^{-08}$	$1,7x10^{-05}$
0048519	negative regulation of biological process	$3,7x10^{-08}$	$1,8x10^{-05}$
0071702	organic substance transport	$4,6x10^{-08}$	$2,1x10^{-05}$
0044248	cellular catabolic process	$4,7x10^{-08}$	$2,1x10^{-05}$
0009987	cellular process	$5,1x10^{-08}$	$2,2x10^{-05}$
0050776	regulation of immune response	$5,5x10^{-08}$	$2,3x10^{-05}$
0033554	cellular response to stress	$6,3x10^{-08}$	$2,5x10^{-05}$
0006629	lipid metabolic process	$6,2x10^{-08}$	$2,6x10^{-05}$
0008202	steroid metabolic process	$6,9x10^{-08}$	$2,7x10^{-05}$
0009056	catabolic process	$8,9x10^{-08}$	$3,4x10^{-05}$
0006955	immune response	$1,1x10^{-07}$	$3,9x10^{-05}$
0044237	cellular metabolic process	$1,4x10^{-07}$	$5,0x10^{-05}$
0042493	response to drug	$1,5x10^{-07}$	$5,2x10^{-05}$
0044699	single-organism process	$1,8x10^{-07}$	$5,8x10^{-05}$

1901698	response to nitrogen compound	$1,7x10^{-07}$	$5,9x10^{-05}$
0007584	response to nutrient	$2,5x10^{-07}$	$8,0x10^{-05}$
0048523	negative regulation of cellular process	$2,5x10^{-07}$	$8,1x10^{-05}$
0097305	response to alcohol	$2,7x10^{-07}$	$8,2x10^{-05}$
0010243	response to organonitrogen compound	$3,1x10^{-07}$	$9,2x10^{-05}$
0015671	oxygen transport	$3,1x10^{-07}$	$9,2x10^{-05}$
